# Understanding the groups of care transition strategies used by U.S. hospitals: an application of factor analytic and latent class methods

**DOI:** 10.1186/s12874-021-01422-7

**Published:** 2021-10-25

**Authors:** Glen Mays, Jing Li, Jessica Miller Clouser, Gaixin Du, Arnold Stromberg, Brian Jack, Huong Q. Nguyen, Mark V. Williams

**Affiliations:** 1 Department of Health Systems, Management and Policy, Colorado School of Public Health, Colorado University, Anschutz, USA; 2 Department of Medicine, Washington University School of Medicine, St. Louis, USA; 3 Center for Health Services Research, College of Medicine, University of Kentucky, Lexington, USA; 4 Department of Statistics, College of Arts and Sciences, University of Kentucky, Lexington, USA; 5 Department of Family Medicine, School of Medicine, Boston University and Boston Medical Center, Boston, USA; 6 Division of Health Services Research and Implementation Science, Kaiser Permanente, Southern California, Pasadena, USA; 7 Division of Hospital Medicine, Washington University School of Medicine, 660 S Euclid Ave; CB 8058, St. Louis, MO 63110 USA

**Keywords:** Transitional care, Comparative effectiveness research, Readmission reduction, Evidence-based practice, Health care

## Abstract

**Background:**

After activation of the Hospital Readmission Reduction Program (HRRP) in 2012, hospitals nationwide experimented broadly with the implementation of Transitional Care (TC) strategies to reduce hospital readmissions. Although numerous evidence-based TC models exist, they are often adapted to local contexts, rendering large-scale evaluation difficult. Little systematic evidence exists about prevailing implementation patterns of TC strategies among hospitals, nor which strategies in which combinations are most effective at improving patient outcomes. We aimed to identify and define combinations of TC strategies, or groups of transitional care activities, implemented among a large and diverse cohort of U.S. hospitals, with the ultimate goal of evaluating their comparative effectiveness.

**Methods:**

We collected implementation data for 13 TC strategies through a nationwide, web-based survey of representatives from short-term acute-care and critical access hospitals (*N* = 370) and obtained Medicare claims data for patients discharged from participating hospitals. TC strategies were grouped separately through factor analysis and latent class analysis.

**Results:**

We observed 348 variations in how hospitals implemented 13 TC strategies, highlighting the diversity of hospitals’ TC strategy implementation. Factor analysis resulted in five overlapping groups of TC strategies, including those characterized by 1) medication reconciliation, 2) shared decision making, 3) identifying high risk patients, 4) care plan, and 5) cross-setting information exchange. We determined that the groups suggested by factor analysis results provided a more logical grouping. Further, groups of TC strategies based on factor analysis performed better than the ones based on latent class analysis in detecting differences in 30-day readmission trends.

**Conclusions:**

U.S. hospitals uniquely combine TC strategies in ways that require further evaluation. Factor analysis provides a logical method for grouping such strategies for comparative effectiveness analysis when the groups are dependent. Our findings provide hospitals and health systems 1) information about what groups of TC strategies are commonly being implemented by hospitals, 2) strengths associated with the factor analysis approach for classifying these groups, and ultimately, 3) information upon which comparative effectiveness trials can be designed. Our results further reveal promising targets for comparative effectiveness analyses, including groups incorporating cross-setting information exchange.

**Supplementary Information:**

The online version contains supplementary material available at 10.1186/s12874-021-01422-7.

## Background

Hospital discharge represents a critical and vulnerable point in the continuum of patient care. As patients transition from hospitals to home or other sites of care, they face myriad challenges arising from a lack of clarity surrounding who is responsible for their care, confusion around complex care plans [1], and often poor communication among health care providers. Unplanned hospital readmissions serve as a marker for poor care transitions resulting in diminished patient satisfaction, increased risk of hospital-acquired infection, and elevated healthcare costs [2, 3]. In an effort to improve the provision of healthcare for Americans, the Hospital Readmission Reduction Program (HRRP) [4], part of the Affordable Care Act, was passed into law in 2010 and activated in 2012. The HRRP reduces payments to hospitals with excess readmissions. As a result, the past decade has seen billions of dollars invested in quality improvement initiatives and value-based payment incentives to improve care transitions and reduce hospital readmissions [5, 6]. Such initiatives include organized transitional care (TC) programs supported by the U.S. Centers for Medicare and Medicaid Services (CMS), including the Hospital Engagement Networks (HENs) [7]—later becoming Healthcare Improvement Innovation Networks (HIINs)— the Quality Improvement Organizations’ Integrating Care for Populations and Communities (QIO ICPC) Aim and the Community-based Care Transitions Programs (CCTPs) [8].

Community and hospital-based TC programs support the implementation of evidence-based models of transitional care, in which bundles of TC services are jointly implemented to meet the diversity of patient needs during care transitions. Some such evidence-based models—e.g., Project RED (Re-Engineering Discharge) [9], Project BOOST (Better Outcomes by Optimizing Safe Transitions) [10], Coleman’s Care Transitions Intervention [11], and Naylor’s Transitional Care Model-TCM [12] – have shown readmission rate reductions ranging from 20 to 40% [9, 12–18]. However, each of these models represents a collection of specific TC strategies (e.g., follow-up appointment with primary care provider), some which overlap among the models, and some of which do not. Faithful implementation of these evidence-based models often varies, with hospitals selectively adapting the component TC services and strategies based on their local context and available resources [19]. Hospitals may provide multiple care transition interventions to patients, selecting components from evidence-based TC models based on staff knowledge and preferences [19, 20]. As a result, the groups of TC strategies that are implemented by hospitals may not accurately reflect evidence-based TC models.

Project ACHIEVE (Achieving Patient-Centered Care and Optimized Health In Care Transitions by Evaluating the Value of Evidence) is a national, observational cohort study to identify the transitional care (TC) outcomes that matter most to patients and family caregivers, and to identify which TC strategies, or types of TC activities, yield desired outcomes for diverse groups of patients and caregivers [21]. Given the dearth of systematic evidence regarding how hospitals group specific TC strategies together, a necessary first step for Project ACHIEVE was to first identify and understand which TC strategies and groups of strategies hospitals use. The purpose of this study is to identify the groups of TC strategies that are implemented among a large and diverse cohort of U.S. short-term acute-care and critical access hospitals, and to characterize the hospitals that choose different combinations based on their institutional, patient and community characteristics.

## Methods

In this analysis, we surveyed hospital representatives about the array of possible TC strategies that hospitals directly implement for patients and their caregivers, along with the services available in the community that hospitals make available to patients and caregivers through referral, linkage, navigation or direct provision of care. This approach provides a view of TC from the hospital perspective, and offers a valuable lens for evaluating hospital-based strategies for managing care transitions. We assume that if a hospital reported implementing a TC strategy to ‘all’ or ‘most’ patients, it appropriately delivered that strategy to all eligible patients.

### Study design

We employed a longitudinal cohort design to study the associations between hospitals’ adoption of groups of TC strategies and their readmission rates during a time in which many hospitals began implementing TC strategies in response to the activation of the HRRP. To assess hospitals’ TC strategy implementation, we conducted a cross-sectional, web-based survey of hospitals’ reported current and previous use of TC strategies from June 2015 through March 2016 in which we asked for specific implementation dates for each TC activity. The American Hospital Association (AHA), America’s Essential Hospitals (AEH) and Joint Commission Resources (JCR) shared the survey link with their membership via REDCap (http://www.redcap.org), a secure, HIPAA-compliant survey administration platform. AHA, AEH, and JCR specifically solicited hospital staff with responsibility for implementing TC in the hospitals.

Respondents reported on the TC efforts in which their organization previously or currently participated. Such efforts included organized TC programs supported by the U.S. CMS--including the HENs, the QIO ICPC Aim and the CCTPs--as well as evidence-based TC models --e.g.,  Project RED [9], Project BOOST [10], Coleman’s CTI [11], and Naylor’s TCM [12]. Survey respondents also indicated whether or not their organization implemented specific TC strategies that are included in established care transition models and/or recommended by professional and scientific organizations.

#### Secondary data sources

Other data sources used in this analysis include are summarized below and described in detail in Additional file 1.

1. Medicare fee-for-service claims data (MEDPAR, inpatient, outpatient, carrier, home health, and SNF Research Identifiable Files), obtained through ResDAC data request;
2. Medicare Master Beneficiary Summary File, obtained through ResDAC request;
3. AHA 2015 Hospital Survey, purchased from AHA;
4. CMS Hospital Impact File, publicly available file;
5. Dartmouth Atlas of Healthcare Health Service Area and Hospital Referral Region data files, publicly available files;
6. Area Health Resources Files (ARHF), publicly available files;
7. Area deprivation index (ADI), publicly available file using 2010 U.S. Census data files.

### Measures

The survey asked respondents to report information on a set of 38 activities nested within 13 cross-cutting TC strategies. The specific activities and strategies included on the survey were identified through reviews of the published peer-reviewed and grey literature on TC programs [22], preliminary findings from focus groups with patients and family caregivers [1] and early phase ACHIEVE site visits [19]. See Additional File 2 for a flow chart of the TC strategy development process. While not an exhaustive inventory of all TC activities used by hospitals, the survey captured information about a core set of practices that are documented in the literature, featured in one or more established TC models, and likely to be observable by hospital personnel involved in patient care and transition planning and management. Survey questions asked whether or not each activity was implemented by the hospital, what types of patients were targeted for the activity, and how frequently the activity was implemented. Seven of the TC strategies included only a single activity, while the remaining six included a combination of 2 or more activities. Some survey response categories were dichotomous (yes/no) while others were ordinal, Likert-type scales. As a result, we constructed dichotomous variables indicating whether or not the hospital implemented these strategies (“yes” being indicated by the most positive responses—e.g., “yes”, “all/most” “always/usually”; and “no” indicated by negative or infrequent responses—e.g., “no”, “sometimes/rarely” “some/none”) for each of the specific 38 activities contained within the 13 TC strategies. We also constructed a composite variable for each of the TC strategies, indicating whether or not the hospital implemented all of the activities nested within it (Additional file 3).

Hospital survey data were linked with claims data for all Medicare fee-for-service beneficiaries admitted to one or more of the responding hospitals between 2010 and 2014 in order to obtain detailed information on patient mix and healthcare utilization trends in each hospital. We define an “episode of care transition” for each inpatient hospital stay as the primary unit of analysis, using a fixed episode that begins with an index hospital admission and ends 30 days after discharge. For each episode we constructed a dichotomous measure of whether an unplanned readmission occurred within 30 days of discharge using the CMS definition of an all-cause unplanned readmission. Any hospital admission that occurred within the 30-day window was classified as a “readmission” rather than a separate index admission. Finally, we measured patient characteristics for each episode including age, gender, race, Medicaid eligibility, the Elixhauser index of comorbidities present prior to and upon admission, and characteristics of the patient’s neighborhood including urban or rural designation, poverty rate, and the Area Deprivation Index for the patient’s zip code area. A total of 3,985,658 patient care transition episodes from 2,369,601 patients are included.

Additionally, hospital survey data were linked with corresponding records from the 2015 American Hospital Association (AHA) annual survey to obtain information on hospital facility characteristics including number of staffed beds, ownership, teaching status, multi-hospital system membership, affiliation with post-acute care providers, and participation in alternative payment models such as accountable care organizations (ACOs) and/or bundled payments. Using the Dartmouth Atlas of Healthcare crosswalk of hospitals to hospital service areas (HSAs) [23], we constructed measures of hospital market structure including the number of short-term acute-care hospitals and hospital beds in each HSA, and the Herfindahl- Hirschman Index (HHI) [24] of hospital market concentration based on hospital discharge volume.

### Statistical analysis

A frequency analysis identified all possible combinations of the 38 TC activities and 13 TC strategies used among the 370 responding hospitals. We performed a Horn’s Parallel Analysis with all 13 TC strategies and 100 random data simulations to determine the number of factors or components to retain [25] (See Additional File 4).Parallel Analysis compares eigenvalues generated from our dataset of TC strategies to eigenvalues generated from random data simulations. We then used exploratory factor analysis to identify a condensed set of TC strategy groups implemented by larger groups of hospitals. We ran the estimated unweighted least squares factor analysis with the 13 dichotomous TC strategy variables, using a polychoric correlation matrix [26] and varimax rotation. The five factors whose eigenvalues exceeded unity were retained as indicators of distinct groups of TC strategies. We assigned a TC strategy as a required element of a TC group (factor) if its factor loading approached or exceeded 0.5, while TC strategies with factor loadings of greater than 0.2 but less than 0.5 were assigned as optional elements of a TC group. Because most hospitals reported implementing more than one of the five TC groups, we classified each hospital into one of 28 mutually exclusive subgroups based on which subsets of the five TC groups they implemented.

As an alternative strategy for identifying distinct groups of TC strategies used by hospitals, we conducted a latent class analysis (LCA) using the 13 TC strategy measures. LCA is a nonparametric statistical method used to identify distinct but partially unobservable subgroups (classes) within a population, based on patterns of response across multiple measures. Unlike factor analysis, LCA identifies classes that are mutually exclusive, and consequently it requires larger sample sizes. We evaluated candidate LCA models specified with 1 to 7 classes based on estimates from Dziak et al. [27] indicating our sample size of 370 had a power of 0.80 to detect 4 classes using 13 measures. We assessed model fit using the Akaike information criterion (AIC) [28], Bayesian information criterion (BIC) [28], and likelihood ratio tests based on Lo-Mendell-Rubin and Vuong-Lo-Mendell-Rubin [29]. When the difference between two models was not statistically significant, we chose the more parsimonious model with fewer classes.

After identifying distinct TC groups using each method, we calculated descriptive statistics to characterize relevant patient, hospital, and community covariates associated with each TC group. Knowing that the activation of the Hospital Readmission Reduction Program in 2012 prompted many hospitals to initiate TC efforts in an effort to reduce readmissions, we assumed that TC implementation began at some point during the study period (2010–2014) and compared readmission rates at the study onset (2010.Q1) to its conclusion (2014.Q3). Therefore, our next step was to estimate mixed-effects regression models using the Medicare patient episode data to test for associations between each TC group and the likelihood of an unplanned hospital readmission within 30 days, while controlling for a rich set of patient, hospital and community covariates. A random-effects specification was used to account for patients clustered within the same hospital, while each TC group was estimated as a fixed effect. To test for differences in readmission trends over time, we estimated the model using data from 2010 to 2014 and added a linear time trend covariate along with interaction terms for each TC group interacted with the time trend. Model results were summarized using average marginal effect estimates from linear probability models rather than odds ratios in order to compare readmission trends across alternative TC groups and alternative models.

## Results

Because our agency partners distributed this public link, we were unable to obtain specific data on response rates. However, as of June 2016, a total of 470 hospital representatives completed the survey. After applying “short-term acute care hospital or critical care hospital” criterion, removing specialty hospitals, and removing duplicate responses from the same hospital and incomplete responses, our final analytic sample consisted of responses from 370 hospitals compared to the 4967 short-term acute-care hospitals in the 2015 AHA data file.^1^ Analysis of these hospitals demonstrated broad diversity with respect to geographic region, urban or rural location, system membership, academic affiliation, and bed capacity, while representing a sample comparative to hospitals across the U.S. (Additional file 5). Small hospitals with fewer than 100 beds were somewhat under-represented among participating hospitals compared to U.S. hospitals overall, while academic medical centers (AMCs) and hospitals with rehabilitation, psychiatric, or skill nursing facility ownership were somewhat over-represented. Geographic location and organizational control of participating hospitals closely matched the U.S. as a whole.

More than 90% of respondents completing the survey characterized their primary role within the hospital as case management, care coordination, social work, or discharge planning, while 8% reported their role as quality improvement or performance management.

### Prevalence of TC strategies

The 370 responding hospitals reported implementing 348 distinct combinations of the 38 TC activities, demonstrating wide variation in practice. Using the 13 composite measures of TC activities, hospitals reported 303 distinct combinations of these strategies. The most prevalent TC strategy, providing patients and caregivers with a written care transition summary at discharge, was used by 78% of hospitals (Table 1). Conversely, only 14% of hospitals reported implementing all of the activities included in a comprehensive needs assessment, making this TC strategy the least prevalent. Hospitals reported implementing a mean of 6.4 of the 13 TC strategies, with 33.8% reporting 8 or more strategies, and 14.6% reporting 3 or fewer strategies (Additional file 6).

**Table 1:** Implementation of Transitional Care Strategies and Activities in U.S. Hospitals

Transitional Care Strategies	Hospitals Implementing (N = 370)
Transition Summary for Patients and Family Caregivers	78.0%
Timely Follow-up	70.5%
Urgent Care Plan	70.5%
Interdisciplinary Approach	69.1%
Transition Team	64.9%
Shared Decisions	61.4%
Referral to Community Services	60.3%
Identify High Risk Patients and Intervene	38.0%
Medication Reconciliation	37.4%
Teach Back	36.1%
Care Coordination	33.7%
Timely Exchange of Critical Patient Information among Providers	22.4%
Patient and Family Caregiver Transitional Care Needs Assessment	14.0%

Note: Sample sizes for individual items range from 362 to 370 due to survey item non-response

We describe TC Strategies as combinations of TC activities as defined in Additional file 2

### Number of factors suggested by parallel analysis

Results from 100 simulations performed in the Horn’s Parallel Analysis (Additional File 4) revealed that the first five factors of the actual data had higher eigenvalues than the first five factors of the simulative data. After the fifth factor, the eigenvalues of the simulative data were overlapped, providing evidence that five factors (i.e., groups of TC strategies) were reasonable.

### TC groups based on factor analysis

Estimated factor loadings from the exploratory factor analysis revealed five distinct groups of TC strategies used by hospitals. Since many TC strategies are closely related, we used factor analysis to identify groups of variables more differentiated, referenced findings from Project ACHIEVE patient and caregiver focus groups [1], and obtained expert clinical and research input to determine the final groups of TC strategies (Table 2). We classified TC strategies as required or optional elements for each group based on the magnitude of factor loadings in combination with expert clinical and research input. The first TC group focused on shared decision making, including required strategy of shared decision-making and optional strategies of needs assessment and teach-back skill assessment. Shared decision making and needs assessment each had factor loadings exceeding 0.5, indicating a relatively strong tendency for hospitals to combine these strategies. A second group focused on targeting high-risk patients, including two required strategies of identifying high risk patients and referring to community services. This group contained only one strategy with a relatively strong factor loading exceeding 0.5. Each of the three remaining TC groups had one strategy with a factor loading approaching but not exceeding 0.5, indicating somewhat weaker and more variable tendencies for hospitals to combine the included strategies. The third group emphasized medication reconciliation activities, while the fourth group focused on care planning and the fifth group centered on cross-setting information exchange between hospital and post-discharge settings.

**Table 2:** Groups of Transitional Care Strategies Based on Factor Analysis Adjusted with Patient and Caregiver Focus Group Findings and Clinical Input

	Estimated Factor Loadings				
Transitional Care Strategies	Factor A (Shared Decision)	Factor B (High Risk)	Factor C (Med Rec)	Factor D (Care Plan)	Factor E (Cross Setting Info Exchange)
1. Urgent Care Plan	0.046	0.071	0.033	**0.465a**	0.018
2. Transition Team	0.144	**0.212b**	**0.318b**	−0.086	**0.236**
3. Care Coordination	0.026	0.086	0.052	**0.304b**	**0.218**
4. Interdisciplinary Approach	−0.007	**0.325b**	**0.233**	−0.185	**0.300**
5. Medication Reconciliation	0.049	−0.101	0.493a^*^	0.083	−0.042
6. Identify High Risk Patients and Intervene	0.023	0.521a^*^	−0.091	0.020	−0.003
7. Patient and Family Caregiver Transitional Care Needs Assessment	**0.511b**	0.055	−0.076	− 0.004	**0.134b**
8. Timely Exchange of Critical Patient Information among Providers	0.022	−0.046	− 0.071	0.084	**0.454a**
9. Referral to Community Services	0.062	0.418a^*^	0.098	0.160	−0.058
10. Shared Decisions	**0.578a**	−0.015	0.085	−0.010	−0.099
11. Teach Back	**0.248b**	0.029	0.006	**0.222**	**0.191b**
12. Timely Follow-up	0.010	0.115	0.396a^*^	0.047	−0.021
13. Transition Summary for Patients and Family Caregivers	−0.092	− 0.002	0.197**b**	**0.267b**	0.118
Total Variance Explained	0.603	0.489	0.516	0.446	0.367

Notes: a. Selected as a required element of factor

a^*^. Either of two elements is required element of factor

b. Selected as an optional element of factor

In total, 91% of responding hospitals reported implementing at least one of the five TC groups, with 71% using two or more of the groups, and 25% using at least four of the groups. The most prevalent group, medication reconciliation, was reported by 72% of hospitals. Less than 16% of hospitals reported using the least prevalent TC group, cross-setting information exchange, but all of these facilities reported using this group together with one or more of the other four TC groups. When hospitals were classified based on which subsets of the five TC groups were used together, a total of 28 mutually exclusive subgroups of hospitals were identified in the sample based on how they implemented TC strategies. Subgroups contained a minimum of 1 and maximum of 62 hospitals.

### TC groups based on LCA

Results from the latent class analysis indicated that best-fit statistics levelled off after 6–7 classes of hospitals were defined in the models. The 7-class model was selected as preferred based on having the highest AIC statistic and based on item-response probabilities indicating best differentiation between classes of hospitals and highest homogeneity within classes. Classes were ordered from high to low based on the number of TC strategies having item-response probabilities greater than 0.5, indicating the spectrum of strategies likely to be used by each class of hospitals. The broadest-spectrum class (Class A), containing 16% of the hospital sample, included 11 strategies with probabilities greater than 0.5 (Table 3). Medication reconciliation and care coordination were the only TC strategies not likely to be implemented by the hospitals in this class. Conversely, the narrowest-spectrum class of hospitals (Class G) included only two TC strategies with probabilities greater than 0.5— urgent care planning and transition summary. More than 20% of the hospitals were grouped into this class, making it the largest class.

**Table 3:** Probability of Implementing Transitional Care Strategies Among Seven Latent Classes of Hospitals

	Hospital Class, Ordered by Spectrum of Strategies Implemented						
	(A)	(B)	(C)	(D)	(E)	(F)	(G)
		Intermediate					
			Shared	Medication	Interdisciplinary	Community	
Transitional Care Strategies	Broad	Care Plan	Decision	Reconciliation	Teams	Services	Narrow
1. Urgent Care Plan	**0.998**	**0.996**	**0.846**	**0.717**	0.043	**0.621**	**0.526**
2. Transition Team	**0.896**	**0.785**	**0.750**	**0.948**	**0.894**	0.484	0.278
3. Care Coordination	0.478	**0.533**	0.169	0.232	0.003	0.002	0.043
4. Interdisciplinary Approach	**0.897**	**0.937**	0.010	**0.995**	**0.995**	**0.692**	0.358
5. Medication Reconciliation	0.288	**0.601**	**0.514**	**0.987**	0.071	0.105	0.214
6. Identify High Risk Patients and Intervene	**0.623**	0.345	0.004	0.389	0.314	0.415	0.026
7. Patient and Family Caregiver Transitional Care Needs Assessment	**0.587**	0.002	0.122	0.153	0.235	0.002	0.002
8. Timely Exchange of Critical Patient Information among Providers	**0.525**	**0.514**	0.151	0.008	**0.523**	0.195	0.155
9. Referral to Community Services	**0.741**	**0.667**	**0.894**	**0.775**	**0.511**	**0.918**	0.061
10. Shared Decisions	**0.998**	0.310	**0.985**	**0.891**	**0.636**	0.450	0.426
11. Teach Back	**0.700**	0.466	**0.509**	0.362	0.264	0.236	0.104
12. Timely Follow-up	**0.822**	**0.860**	**0.622**	**0.960**	**0.627**	**0.636**	0.488
13. Transition Summary for Patients and Family Caregivers	**0.831**	**0.994**	**0.986**	**0.736**	**0.992**	**0.653**	**0.524**
Percent of hospitals in class	16.2%	13.5%	8.4%	13.5%	9.5%	17.3%	21.6%
Number of hospitals in class	60	50	31	50	35	64	80
Number of TC strategies with *p* > 0.5	11	9	8	8	7	5	2
Entropy	0.81						

Note: Estimates were obtained from a latent class model with 7 classes. Bold indicates item response probabilities> 0.5

The remaining five classes ranked as intermediate-spectrum classes, each having between 5 and 8 TC strategies with probabilities exceeding 0.5. In contrast to the broad-spectrum class, all five intermediate classes were unlikely to identify high-risk patients and use needs assessment. The broadest of the intermediate classes comprised hospitals that were unique in their tendency to use care coordination along with medication reconciliation and cross-setting information exchange. A second intermediate class contained hospitals that were likely to use medication reconciliation and teach-back techniques, while a third intermediate class was distinctive in using medication reconciliation without teach-back or cross-setting information exchange strategies. The fourth and fifth intermediate classes were distinctive in being unlikely to use medication reconciliation but likely to use interdisciplinary teams and referral to community services, in combination with selected other strategies.

The seven classes of hospitals identified through LCA analysis corresponded closely with selected groups of TC strategies identified through factor analysis. Hospitals included in the broad-spectrum LCA class, for example, were likely to implement all five of the factor analysis groups. Hospitals classified into each of the intermediate LCA classes were likely to implement between two and four of the factor analysis groups. By contrast, hospitals classified into the narrow LCA class were unlikely to implement any of the factor analysis groups.

### Differences in hospital readmission trends across TC groups

Among fee-for-service Medicare beneficiaries discharged from all 370 study hospitals, the unplanned 30-day readmission rate declined from 15.3% in 2010 to 14.4% in 2014. When hospitals were classified based on their use of the five non-exclusive TC groups, regression estimates indicated that three TC groups were associated with reductions in the risk of a 30-day readmission that significantly exceeded the reductions observed among the reference group of hospitals that used none of the TC groups (Table 4, Model 1). The largest reduction in readmission risk occurred among hospitals that used the *Cross-setting Information Exchange* TC group, resulting in an average reduction of 0.60 percentage-points per year after adjusting for patient, hospital and community characteristics (*p* < 0.01). Hospitals using the *Shared Decision* group of TC strategies were estimated to achieve an annual reduction of 0.56 percentage-points (*p* = 0.01), while those using the *Care Plan* TC strategy group were estimated to achieve a 0.54 percentage-point annual reduction (*p* = 0.05).

**Table 4:** Associations Between Transitional Care Combinations and Trends in 30-Day Readmissions

	Risk-Adjusted Change in Probability of 30-day		
Transitional Care Combination	Readmission, 2010–14		
	Estimate	St. Error	*P* value*
**Model 1: Non-Exclusive Combinations Based on Factor Analysis**			
Factor A: Shared Decision	**−0.0056**	**0.0006**	**0.0127**
Factor B: Identify High Risk	− 0.0046	0.0006	0.2989
Factor C: Medication Reconciliation	−0.0053	0.0006	0.1948
Factor D: Care Plan	**−0.0054**	**0.0006**	**0.0521**
Factor E: Cross-Setting Information Exchange	**−0.0060**	**0.0006**	**0.0040**
No Factor Used (reference)	−0.0049	0.0003	
**Model 2: Mutually Exclusive Combinations Based on Factor Analysis (ordered by magnitude)**			
Factors A + B + C + E	**−0.0106**	**0.0017**	**0.0001**
Factors A + C + D + E	**−0.0096**	**0.0024**	**0.0149**
Factors B + C + D + E	**−0.0089**	**0.0020**	**0.0117**
Factors B + C + E	**−0.0080**	**0.0014**	**0.0007**
Factors A + B	−0.0071	0.0030	0.3891
Factors A + B + D	−0.0070	0.0016	0.0665
Factors A + B + D + E	−0.0070	0.0016	0.0870
Factors A + B + C + D + E	**−0.0070**	**0.0012**	**0.0033**
Factors A + B + C + D	**−0.0066**	**0.0011**	**0.0093**
Factors C + D	**−0.0065**	**0.0011**	**0.0209**
Factors A + C + D	**−0.0065**	**0.0010**	**0.0088**
Factor A alone	**−0.0064**	**0.0011**	**0.0317**
Factor D alone	−0.0064	0.0014	0.1272
Factors A + B + C	−0.0059	0.0016	0.3755
Factors B + E	−0.0058	0.0016	0.4562
Factors A + C	−0.0058	0.0011	0.1960
Factors B + D	−0.0056	0.0017	0.6053
Factors A + D	−0.0055	0.0012	0.3749
Factor C alone	−0.0054	0.0011	0.4658
Factors D + E	−0.0048	0.0019	0.9495
Factors B + D + E	−0.0048	0.0021	0.9340
Factors C + E	−0.0041	0.0038	0.8056
Factors B + C + D	−0.0040	0.0014	0.3056
Factors C + D + E	−0.0033	0.0023	0.3752
Factor B alone	−0.0032	0.0024	0.3514
Factors A + D + E	−0.0017	0.0018	0.1180
Factors B + C	**0.0014**	**0.0015**	**0.0001**
No Factors Used (reference)	−0.0049	0.0005	
**Model 3: Mutually Exclusive Combinations Based on Latent Class Analysis**			
Class A: Broad Spectrum	**−0.0031**	**0.0013**	**0.0192**
Class B: Intermediate with Care Plan	−0.0057	0.0011	0.9680
Class C: Intermediate with Shared Decision	**−0.0100**	**0.0010**	**0.0001**
Class D: Intermediate with Medication Reconciliation	**−0.0025**	**0.0009**	**0.0001**
Class E: Intermediate with Interdisciplinary Teams	−0.0061	0.0008	0.5966
Class F: Intermediate with Referral to Community Services	**−0.0071**	**0.0005**	**0.0006**
Class G: Narrow Spectrum (Reference)	−0.0058	0.0001	
**Model 4: Hospital random effects and time trend only**			
Full sample	**−0.0028**	**0.0001**	**0.0010**
**Model Diagnostics**	-2Ln(L)	AIC	BIC
Model 1	2,916,257	2,916,261	2,916,268
Model 2	2,916,684	2,916,688	2,916,695
Model 3	2,916,261	2,916,265	2,916,273
Model 4	3,049,854	3,049,858	3,049,866

Note: Estimates are from linear probability models with random hospital effects, adjusting for age, sex, race, dual-eligibility status, disability status, Elixhauser comorbidities, Medicare expenditures in past 180 days, urban/rural designation of hospital, Area Deprivation Index, Health Professions Shortage Area designation, teaching hospital designation, hospital ownership type, and monthly time trend

Mutually exclusive TC combinations are defined such that each hospital is classified into exactly one combination

*P-value indicates the significance of the difference between the coefficient estimate for the current category and the reference category in each model. Bold text indicates p < 0.05

When hospitals were classified into 28 mutually exclusive subgroups based on factor analysis TC groups, regression estimates indicated that the largest reductions in readmission risk occurred among hospitals that used the *Cross-setting Information Exchange* TC strategy group together with at least two additional TC groups (Table 4, Model 2). A total of 9 hospital subgroups (33%) were associated with reductions in readmission risk that significantly exceeded the trend observed among the reference group of hospitals that used no TC combinations (*p* < 0.05), after adjusting for patient, hospital and community characteristics. Of these 9 subgroups with significant readmission trend differences, 5 subgroups (56%) involved the *Cross-setting Information Exchange* and *Medication Reconciliation* TC groups along with at least one additional TC group. The 4 remaining subgroups with significant trend differences all involved the Care Plan TC strategy group, and 3 of these subgroups also involved *Medication Reconciliation*.

When hospitals were organized into the 7 mutually-exclusive classes based on latent class analysis, regression estimates indicated that the largest reductions in readmissions occurred among hospitals using two of the intermediate-spectrum TC classes–one class emphasizing shared decision-making (Class C), and the other class emphasizing referrals to community services (Class F) (Table 4, Model 3). Counterintuitively, hospitals using the broad-spectrum TC class (Class A) and the intermediate-spectrum class (Class D) emphasizing medication reconciliation were estimated to achieve significantly smaller reductions in readmission than the reference group of hospitals using the narrow-spectrum TC class, after adjusting for patient, hospital and community characteristics (*p* < 0.05).

Model specification tests using AIC, BIC, and J statistics indicated that TC groups based on factor analysis (model 1) performed better than the ones based on latent class analysis (model 3) in detecting differences in 30-day readmission trends (Table 4).

## Discussion

For over a decade after the Affordable Care Act was passed, hospitals have engaged in widespread experimentation regarding their implementation of TC strategies in an effort to reduce unnecessary hospital readmissions. While many hospitals implement specific evidence-based TC models, adaptation of the TC strategies included in those models is prevalent, rendering comparative effectiveness trials difficult. The present study sought to apply novel methods to group TC strategies for evaluation purposes in order to uncover current practice patterns in TC strategy implementation. Through this process we found 1) wide variation in the combinations of TC strategies used by U.S. hospitals to manage care transitions among hospitalized patients, and 2) Factor analysis provides a logical method for grouping TC strategies when the groups are dependent and overlapping.

By focusing on strategy groups rather than individual TC strategies, we found that practice variation among hospitals is more heterogeneous than suggested by previous studies. Further, our findings add value to the TC literature because the groupings suggested through our analysis reveal hospitals’ actual implementation efforts compared to conceptual frameworks and models that may not be implemented with fidelity. Further analyses revealed that patient demographic, socioeconomic, and diagnostic characteristics were not strongly associated with the specific combinations of TC strategies used by hospitals in our study, suggesting that case mix is unlikely to be a major determinant of hospitals’ TC choices. Other factors, such as staff knowledge and preferences, implementation costs, and accumulated experiences with implementation might be a more important factor in the choice of TC strategy.

The extent of practice variation revealed in this study underscores the need to identify feasible approaches to classify hospitals based on the type of TC strategies they choose to implement, which leads to our second key finding. Our results demonstrate that TC strategy classification based on factor analysis was better able to detect differential trends in 30-day readmission rates compared to TC strategy classification based on latent class analysis. One reason for the superior performance of factor analysis is its lack of dependence on a constraint requiring mutually exclusive assignment of hospitals to TC groups, thereby allowing greater flexibility in grouping related TC strategies. This non-exclusive approach to classification, however, makes the factor analysis method less straightforward to interpret when used in comparative analyses because many individual hospitals may be assigned to multiple TC groups. Nonetheless, the reality of non-exclusivity in implementation of transitional care strategies is part of the initial rationale for this study: hospitals, in reality, implement groups of TC strategies in diverse combinations that overlap with one another. As a result, an analytic approach to grouping transitional care strategies that recognizes and accounts for that overlap is preferred. We tested a feasible solution to this non-exclusivity problem that entails using factor analysis results to construct a more expansive set of classifications comprising 28 unique combinations of TC groups. This enhanced classification approach provided a detailed view of the TC groups most strongly associated with trends in hospital readmissions when used in a mixed-effects regression model framework. As such, this enhanced factor analysis approach represents our preferred method for TC classification and comparison.

Our ultimate preference for the FA over LCA for classifying groups of TC strategies occurs in light of the strengths and limitations we recognize for each method. Factor analysis is appropriately used when constructs included in the analysis are reflective in nature, while latent class analysis is more appropriate when constructs are formative (i.e., latent) [30]. In other words, performing factor analysis as a method for classifying groups assumes all relevant TC strategies have been measured and included as constructs, whereas latent class analysis assumes there may be unmeasured constructs (formative constructs) influencing hospitals’ implementation patterns. Importantly, prior to the present study, we performed a multistep process [22] to develop and define the 13 included TC strategies based on available evidence and review/adjudication by a diverse group of experts and stakeholders in the care transitions field. As a result, we feel confident about the relevance of the TC strategies included in our analysis and their treatment as reflective constructs. Nonetheless, with the recognition that there may be some unmeasured components that our process did not account for, we applied LCA to help anchor the FA results and explore if new insights emerged that may influence our approach to grouping. Our results reveal promising targets for implementing groups of TC strategies. The substantive findings from our preferred classification method indicated that hospitals with better sharing of health information with sources of ongoing care (i.e., the *Cross-setting Information Exchang*e TC group), when used together with two or more additional TC groups, uniquely identified hospitals with the largest downward trends in readmissions. Groups involving *Cross-setting Information Exchange* paired with hospitals providing care plans and documenting medication reconciliation (i.e. the *Care Plan* and/or *Medication Reconciliatio*n strategy) showed downward trends in hospital readmissions that were statistically significant. These groups of TC strategies, used by more than 20% of the hospitals, represent promising interventions in future comparative effectiveness studies that compare strategic approaches to readmission reduction.

Results from this study should be interpreted with caution in view of several important limitations. TC strategies were self-reported by a single hospital staff and may not reflect actual practices with high levels of precision. Attempting to mitigate the potential for self-report bias regarding TC strategy implementation, we used data collected through the study’s Phase 1 hospital site visits [19] to confirm survey results among four hospitals that participated in both study components. We found no inconsistencies in TC effort reporting and implementation through this process; therefore, we feel confident in the survey’s validity as a measure of TC strategy implementation. Another limitation is the potential for selection bias. Although our sample showed diversity across several important characteristics (e.g., geography, rurality), it was not nationally representative (e.g., it included a higher proportion of larger hospitals with ownership of SNFs, rehab facilities), which may influence the resources available to participating hospitals to invest in TC strategies.

Some important TC strategies used by hospitals may not have been included on our survey and therefore may be omitted from study. Further, because this approach offers the view of TC from the hospital perspective, it may not provide a complete view of all the TC services or resources available to a patient and caregiver in a given community, therefore may not completely identify the TC strategies actually received and used by a given patient and caregiver. In addition, many hospitals did not report detailed information about the time frames in which TC strategies were implemented, thereby limiting the study’s ability to evaluate temporal relationships between TC strategies and readmission trends. However, activation of the Hospital Readmission Reduction Program in 2012 prompted many hospitals to initiate TC efforts in an effort to reduce readmissions. Therefore, to address the problem of missing implementation dates, we assumed that TC implementation began at some point during the study period (2010–2014) and compared readmission rates at the study onset (2010.Q1) to its conclusion (2014.Q3). Due to the observational design of this study, the estimated associations between groups of TC strategies and readmission trends should not be interpreted as causal relationships and may be influenced by unmeasured patient, hospital, and community characteristics as well as unrelated temporal trends. Nevertheless, this study’s findings provide preliminary support for a method of classifying and comparing hospitals based on clinically relevant differences in the content of their TC strategies.

## Supplementary Information

**Additional file 1.** Retrospective Study Model Covariates and Data Sources.

**Additional file 2.** Flow Chart of TC Strategy Development Process.

**Additional file 3.** Implementation of Transitional Care Strategies and Required Activities in U.S. Hospitals.

**Additional file 4.** Horn’s Parallel Analysis Results.

**Additional File 5.** Characteristics of Participating Hospitals.

**Additional file 6.** Frequency of TC Strategy Adoption.

## Data Availability

Medicare data are prohibited from being shared, so we will be unable to share the Medicare claims data that were purchased for analysis. However, cleaned, complete, and de-identified primary data collected from this study will be made available. For the purposes of sharing and disseminating the research data, all direct identifiers and any indirect identifiers that could be used in conjunction with other publicly available information to identify individuals will be removed from the study data. Quantitative data will be organized into the structure of a portable SAS file with all data clearly defined. We will make the data and associated documentation available to users under a data-sharing agreement that provides for: [1] a commitment to using the data only for research purposes and not to identify any individual participant [2]; a commitment to securing the data using appropriate methodology and technology; and [3] a commitment to destroying or returning the data after analyses are completed. Should other researchers in the U.S. request our data, we will be able to transmit it to them via a HIPAA secure website.

## Abbreviations

ACHIEVE: Achieving Patient-Centered Care and Optimized Health In Care Transitions by Evaluating the Value of Evidence
TC: Transitional care
HRRP: Hospital Readmission Reduction Program
CMS: Centers for Medicare and Medicaid Services
HENs: Hospital Engagement Networks
HIINs: Healthcare Improvement Innovation Networks
QIO ICPC: Quality Improvement Organizations’ Integrating Care for Populations and Communities
CCTPs: Community-based Care Transitions Programs
Project BOOST: Better Outcomes by Optimizing Safe Transitions
Project RED: Re-Engineering Discharge
CTI: Care Transitions Intervention
TCM: Transitional Care Model
AHA: American Hospital Association
JCR: Joint Commission Resources
AEH: America’s Essential Hospitals
ACOs: Accountable Care Organizations
HSAs: Hospital Service Areas
HHI: Herfindahl- Hirschman Index
LCA: Latent class analysis
AIC: Akaike information criterion
BIC: Bayesian information criterion
AMCs: Academic Medical Centers
PCORI: Patient-Centered Outcomes Research Institute
U.S.: United States

## Footnotes

1 If more than one response was received from a single hospital, the most complete response was retained. If two or more records with similar completion rates were received from the same hospital, we retained the record completed by the participant with the most direct knowledge of TC efforts (e.g, care coordinator, case manager, discharge coordinator).
