# Supplementary material for: Understanding the groups of care transition strategies used by U.S. hospitals: an application of factor analytic and latent class methods
Source: BMC Med Res Methodol. 2021 Oct 25;21:228. doi: 10.1186/s12874-021-01422-7 (PMC8543851; doi:10.1186/s12874-021-01422-7)
Supplement: Supplementary file 1 — Additional file 1. Retrospective Study Model Covariates and Data Sources. [file 12874_2021_1422_MOESM1_ESM.docx]

| **Retrospective Study Model Covariates and Data Sources** |  |
| --- | --- |
| **Covariate List** | **Sources** |
| *Patient level*   - Age - Race - Gender - Previous 12 months Part D covered - Rural/urban (patient zipcode) - Reason for Medicare eligibility (age, End Stage Renal Disease, etc.) - Dual-eligible status at admission - 25 Elixhauser comorbidities (e.g., chronic disease, congestive heart failure, peripheral vascular disease, etc.) - Total number of Elixhauser comorbidities - HCC cohort (e.g., cardiovascular, medicine, surgery, neurology, cardiorespiratory) - Discharge disposition status (home without home health, SNF, inpatient rehab, long-term acute care, hospice, home with home health, other) - Number of prior physician encounters within prior 6 months - Number of inpatient stays in prior 6 months - Total inpatient days in prior 6 months - Number of HHA stays in prior 6 months - Number of SNF stays in prior 6 months - Total expenditure of prior inpatient stays in prior 6 months - Patient distance from admitting hospital - Patient distance from nearest hospital | *Medicare Claims Data* |
| *Hospital level covariates*   - Bed size - Academic medical center (AMC) | *American Hospital Association 2015 Hospital Survey* |
| *Hospital level covariates*   - For-profit status - Teaching-center status - Participation in alternative payment models (e.g., ACO, bundle payment) - Hospital system membership - Hospital Structure (e.g., owning rehabilitation service, SNF, home health, LTAC, palliative care, hospice) - Enabling services (e.g. Case management, geriatric services, patient education center, etc.) | *FY2018 CMS Hospital Impact File* |
| - HHI hospital competition index | *Dartmouth Atlas of Healthcare Health Service Area Hospital Referral Region data files* |
| *Community-level covariates based on patient county of residence*   - Urban/rural - Skilled nursing facility (SNF) beds per 100,000 residents - Primary care providers (PCP) per 100,000 residents - Hospital beds per 100,000 residents - Percentage with health insurance <65 - Annual median household income - Annual high school completion rate - Annual % population nonwhite - Annual proportion population below 100% poverty level | *Area Health Resources Files (ARHF)* |
| - Area Deprivation Index (ADI) | *2010 U.S. Census data* |
| **Data Source Description** | |
| **Medicare Fee-For-Service (FFS) Claims Data**  Medicare claims data represent a convenience sample that includes approximately 30 million individuals annually. The FFS claims data used in this study contains data for each county broken out by aged, disabled, and ESRD beneficiaries and includes data on total Medicare FFS reimbursement and enrollment for Parts A and B; the corresponding per capita reimbursement; for Part A, reimbursement for direct (DME) and indirect medical education (IME) expenditures and disproportionate share expenditures (DSH); and the per capita expenditures with the medical education and disproportionate share expenditures removed.  Through ResDAC, project ACHIEVE obtained access to CMS research identifiable files (RIFs), including: MEDPAR, inpatient, outpatient, carrier, home health, and SNF Research Identifiable Files.  **Medicare Master Beneficiary Summary File (MBSF)**  There are 4 segments to the MBSF ˗ 1) Beneficiary Summary File or Medicare Enrollment (A/B/C/D information), 2) Chronic Conditions, 3) Cost & Utilization, and 3) NDI Death Information (includes ICD-10 Cause of Death). The file includes variables such as State/county annual SSA codes, State/county monthly FIPS codes, Zip code, State and County, Date of birth, date of death, Race, Reason for entitlement, Monthly enrollment for each part of the Medicare program, A/B/C/D, Dual eligible status, Part C plan and enrollment information, Part D plan and enrollment information, Part D low income cost sharing. Medicare FFS Claims data and MBSF were purchased through ResDAC.  **American Hospital Association (AHA) Hospital Survey File**  AHA collects data directly from nearly 6,300 hospitals and more than 400 health care systems, including demographics, operations, service line, staffing, c-suite information, expenses, physician organization structures, beds, utilization and more. AHA hospital survey file was purchased from AHA.  **CMS Hospital Impact File**  The CMS hospital impact file is a publicly available file prepared in the summer preceding the Federal Fiscal year and are based on the best data available at the time. The files are used in estimating payment impacts of various policy changes to the Inpatient Prospective Payment System (IPPS) proposed.  **Dartmouth Atlas of Healthcare Health Service Area and Hospital Referral Region data files**  These files use a methodology, commonly known as small area analysis, which is population-based. The focus of small area analysis is on the experience of the population living in a defined geographic area or the population that uses a specific hospital. These files provide data on hospital market (e.g., number of hospital beds) in hospital service areas (HSA), or a collection of ZIP codes whose residents receive most of their hospitalizations from the hospitals in that area. HSAs were defined by assigning ZIP codes to the hospital area where the greatest proportion of their Medicare residents were hospitalized. Hospital referral regions (HRRs) represent regional health care markets for tertiary medical care; each containing at least one hospital performing major cardiovascular procedures and neurosurgery.  **Area Health Resources Files (ARHF)**  The AHRF is a publicly available file that include data collected from more than 50 sources with over 6,000 variables related to health care access at the county level. Its data include information on geographic codes and classifications; health professions supply and demographics; health facility numbers and types; hospital utilization; population characteristics and economic data; land use and housing density; and health professions training resources.  **Area deprivation index (ADI)**  The ADI represents a geographic area-based measure of the socioeconomic deprivation experienced by a neighborhood. Higher index values represent higher levels of deprivation, which have been associated with an increased risk of adverse health and health care outcomes. ADI was publicly obtained using the 2010 U.S. Census data files. | |
