# Supplementary figures and images for: Understanding the groups of care transition strategies used by U.S. hospitals: an application of factor analytic and latent class methods
Source: BMC Med Res Methodol. 2021 Oct 25;21:228. doi: 10.1186/s12874-021-01422-7 (PMC8543851; doi:10.1186/s12874-021-01422-7)

Additional File 2. TC Strategy Development Flow Chart


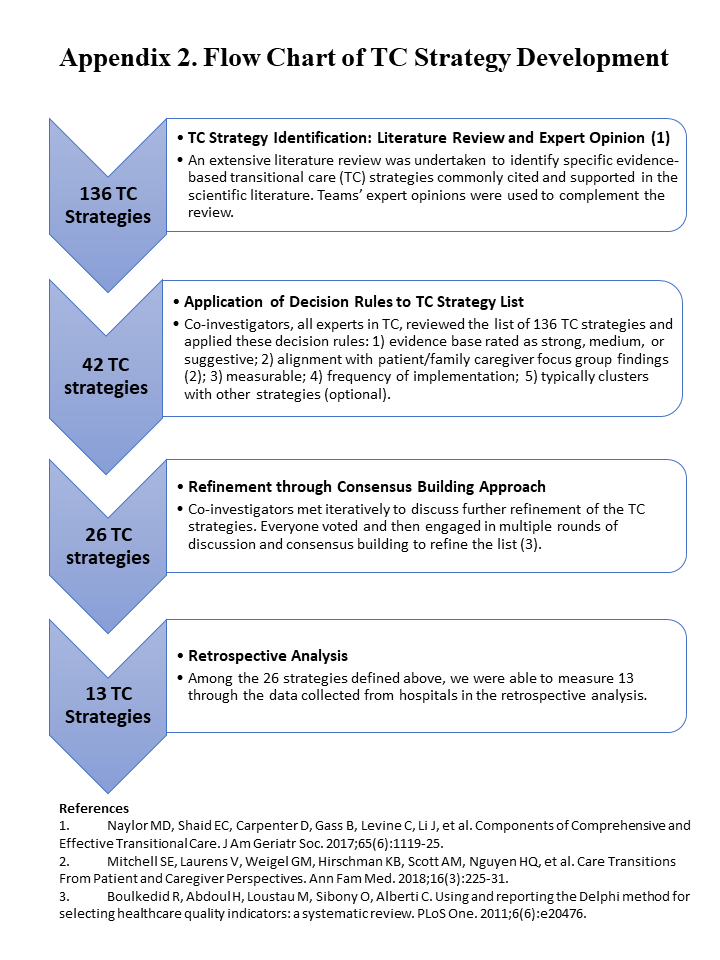

Supplement: Supplementary file 2 — Additional file 2. Flow Chart of TC Strategy Development Process. [file 12874_2021_1422_MOESM2_ESM.docx]
