# Supplementary material for: Understanding the groups of care transition strategies used by U.S. hospitals: an application of factor analytic and latent class methods
Source: BMC Med Res Methodol. 2021 Oct 25;21:228. doi: 10.1186/s12874-021-01422-7 (PMC8543851; doi:10.1186/s12874-021-01422-7)
Supplement: Supplementary file 3 — Additional file 3. Implementation of Transitional Care Strategies and Required Activities in U.S. Hospitals. [file 12874_2021_1422_MOESM3_ESM.docx]

| **Table 1. Implementation of Transitional Care Strategies and Required Activities in U.S. Hospitals** | |
| --- | --- |
| **Strategy 1: Urgent Care Plan.** Hospital implements both activities 1.1 and 1.2 below | |
| 1.1 | At time of discharge, provide all or most patients a written list of the signs and symptoms that should prompt an immediate call to PCP or a return to the hospital |
| 1.2 | At time of discharge, provide all or most patients written direct contact information for the provider to be contacted for an urgent or emergent issue |
| **Strategy 2: Transition Team.** Hospital implements activity 2.1 below | |
| 2.1 | Routinely use a specific transition team to coordinate transition plans across hospital and post-acute sites of care |
| **Strategy 3: Care Coordination.** Hospital implements all activities 3.1 through 3.7 below | |
| 3.1 | Routinely call patients within 1 week after discharge to follow up on post-discharge needs |
| 3.2 | Routinely assign someone to follow up on test results that arrive after a patient is discharged |
| 3.3 | At time of discharge, provide all or most patients with a written action plan to help patients and caregivers manage changes in their condition |
| 3.4 | At time of discharge, provide all or most patients with a written description of the names, doses, frequencies, and purpose of each prescribed medication |
| 3.5 | At time of discharge, provide all or most patients with a written description of medications that were newly added, changed and discontinued during the hospital stay |
| 3.6 | For patients discharged to a skilled nursing facility, always or usually conduct a nurse-to-nurse report prior to discharge |
| 3.7 | For patients discharged to a skilled nursing facility, always or usually provide the facility with a direct contact number to reach the inpatient treating physician |
| **Strategy 4: Interdisciplinary Approach.** Hospital implements activity 4.1 below | |
| 4.1 | Use a multidisciplinary team to manage the care of patients at high risk of readmission |
| **Strategy 5: Medication Reconciliation.** Hospital implements both activities 5.1 and 5.2 below | |
| 5.1 | Always or usually contact outside pharmacies or primary care providers to clarify the patient's current medication list |
| 5.2 | Formally designate a physician, physician assistant, nurse, or pharmacist to conduct medication reconciliation at discharge |
| **Strategy 6: Identify High Risk Patients and Intervene.** Hospital implements all activities 6.1-6.3 and at least 4 of activities 6.4-6.10 | |
| 6.1 | Always or somewhat consistently use a protocol to identify patients who are at high risk of readmission |
| 6.2 | Estimate each patient's risk of readmission in a formal way and use the estimate in clinical care |
| 6.3 | Always or somewhat consistently implement risk-specific interventions tailored to each patient's individual risk of readmission or other post-discharge adverse event |
| 6.4 | Identify patients who will receive transitional care services based on patient diagnoses |
| 6.5 | Identify patients who will receive transitional care services based on availability of social support |
| 6.6 | Identify patients who will receive transitional care services based on difficulties taking medications as prescribed |
| 6.7 | Identify patients who will receive transitional care services based on health literacy |
| 6.8 | Identify patients who will receive transitional care services based on hospital or ED use in past 30 days |
| 6.9 | Identify patients who will receive transitional care services based on cognitive function |
| 6.10 | Identify patients who will receive transitional care services based on emotional/psychological status |
| **Strategy 7: Patient and Family Caregiver Transitional Care Needs Assessment.** Hospital implements all activities 7.1-7.5 below | |
| 7.1 | Assess each patient's transitional care needs |
| 7.2 | Assess each family caregiver's transitional care needs |
| 7.3 | Implement comprehensive transitional care planning |
| 7.4 | Routinely ask whether patients can afford their medications as part of discharge process |
| 7.5 | Screen all patients to identify post-discharge needs using explicit criteria |
| **Strategy 8: Timely Exchange of Critical Patient Information among Providers.** Hospital implements all activities 8.1-8.4 below | |
| 8.1 | Ensure outpatient providers are alerted to patient's admission within 24 hours |
| 8.2 | Complete discharge summary and make available for viewing within 72 hours of discharge |
| 8.3 | Send discharge summary directly to patient's PCP for all or most patients |
| 8.4 | Ensure that patient's outpatient providers have access to inpatient medical records for all or most patients |
| **Strategy 9: Referral to Community Services.** Hospital implements activity 9.1 below | |
| 9.1 | Refer patients to community-based services to a great extent or somewhat routinely |
| **Strategy 10: Shared Decisions.** Hospital implements activity 10.1 below | |
| 10.1 | Engage patients and family caregivers in decision-making about transitional care |
| **Strategy 11: Teach Back.** Hospital implements activity 11.1 below | |
| 11.1 | Use teach-back techniques for patient and family education to a great extent or somewhat routinely |
| **Strategy 12: Timely Follow-up.** Hospital implements activity 12.1 below | |
| 12.1 | Always or usually ensure patients leave the hospital with outpatient follow-up appointment already arranged |
| **Strategy 13: Transition Summary for Patients and Family Caregivers.** Hospital implements activity 13.1 below | |
| 13.1 | Provide all or most patients and family caregivers with a personal health record in written form at time of discharge, including list of diagnoses, allergies, medications, and physician contact information |
