# Supplementary material for: Understanding the groups of care transition strategies used by U.S. hospitals: an application of factor analytic and latent class methods
Source: BMC Med Res Methodol. 2021 Oct 25;21:228. doi: 10.1186/s12874-021-01422-7 (PMC8543851; doi:10.1186/s12874-021-01422-7)

Additional File 4. Results from Horn’s Parallel Analysis

In performing 100 simulations, we found that five factors (i.e., components) was optimal.


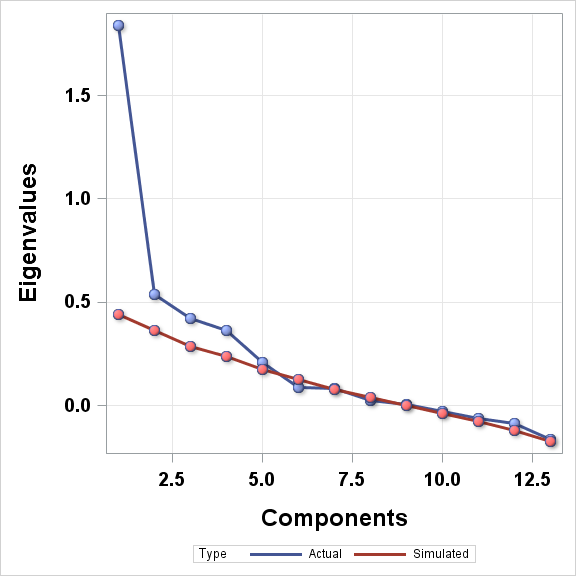

Supplement: Supplementary file 4 — Additional file 4. Horn’s Parallel Analysis Results. [file 12874_2021_1422_MOESM4_ESM.docx]
