# Supplementary material for: Understanding the groups of care transition strategies used by U.S. hospitals: an application of factor analytic and latent class methods
Source: BMC Med Res Methodol. 2021 Oct 25;21:228. doi: 10.1186/s12874-021-01422-7 (PMC8543851; doi:10.1186/s12874-021-01422-7)
Supplement: Supplementary file 5 — Additional File 5. Characteristics of Participating Hospitals. [file 12874_2021_1422_MOESM5_ESM.docx]

| **Characteristics of Participating Hospitals** | | | | |
| --- | --- | --- | --- | --- |
|  | **Hospital Characteristics** | **ACHIEVE Survey**  **(N=370)^*^** | **US**  **(N=4967)^**^** | |
|  | **Region** |  |  | |
|  | Midwest | 94(25.40) | 1413(28.4) | |
|  | Northeast | 49(13.20) | 582(11.7) | |
|  | South | 169(45.70) | 1801(36.3) | |
|  | West | 49(13.20) | 926(18.6) | |
|  | Puerto Rico | 9(2.40) | 51(1.0) | |
|  | Unknown | 0(0) | 194 (3.9) | |
|  | **Urban/Rural ^b^** |  |  | |
|  | Rural | 148(40.0) | 1445(29.1) | |
|  | Urban | 222(60.0) | 3522(70.9) | |
|  | **Organizational Control** |  |  | |
|  | For profit | 44(11.9) | 892 (15.2) | |
|  | Gov, federal | 1(0.3) | 35(0.7) | |
|  | Gov, nonfederal | 93(25.1) | 1040(20.9) | |
|  | Not-for-profit | 232(62.7) | 2809(56.6) | |
|  | Unknown | 0(0) | 191(3.8) | |
|  | **AMC** |  |  | |
|  | Yes | 45(12.2) | 251(5.1) | |
|  | No | 316(85.4) | 4267(85.9) | |
|  | Unknown | 9(2.4) | 449(9.0) | |
|  | **Total Licensed Beds** |  |  | |
|  | <100 | 122(33.0) | 2300(46.3) | |
|  | 100-299 | 138(37.3) | 1460(29.4) | |
|  | >=300 | 101(27.3) | 760(15.3) | |
|  | Unknown | 9(2.4) | 449(9.0) | |
|  | **Type of Units** |  |  | |
|  | Rehabilitation | 99(26.80) | 804(16.2) | |
|  | Psychiatric | 145(39.20) | 1130(22.8) | |
|  | Skilled Nursing | 70(18.90) | 723(14.6) | |
|  | Nursing Home/LTAC | 3(0.80) | 38(0.8) | |
|  | **Programs** |  |  | |
|  | PCMH^c^ | 74(20.00) | 743(15.0) | |
|  | Palliative Care | 45(12.20) | 403(8.1) | |
|  | Hospice | 88(23.80) | 877(17.7) | |
| Sources:  * Hospitals responding to the Project ACHIEVE Hospital Adoption Survey (2015-2016) **American Hospital Association 2015 Annual Survey. U.S. estimates are for facilities classified as short-term or critical access hospitals. | | | |  |
