# Supplementary material for: Understanding the groups of care transition strategies used by U.S. hospitals: an application of factor analytic and latent class methods
Source: BMC Med Res Methodol. 2021 Oct 25;21:228. doi: 10.1186/s12874-021-01422-7 (PMC8543851; doi:10.1186/s12874-021-01422-7)
Supplement: Supplementary file 6 — Additional file 6. Frequency of TC Strategy Adoption. [file 12874_2021_1422_MOESM6_ESM.docx]

| **Frequency of TC Strategy Adoption** | | |
| --- | --- | --- |
| **TC Strategies Applied** | **Hospitals** | |
| **Number** | **N** | **%** |
| **0** | 3 | 0.81 |
| **1** | 8 | 2.16 |
| **2** | 18 | 4.86 |
| **3** | 25 | 6.76 |
| **4** | 40 | 10.81 |
| **5** | 39 | 10.54 |
| **6** | 50 | 13.51 |
| **7** | 62 | 16.76 |
| **8** | 46 | 12.43 |
| **9** | 37 | 10.00 |
| **10** | 27 | 7.30 |
| **11** | 8 | 2.16 |
| **12** | 6 | 1.62 |
| **13** | 1 | 0.27 |
